# Supplementary material for: Are carbon emissions trading and green financial instruments synergistic? -Comprehensive quantitative research based on content analysis
Source: PLoS One. 2024 Mar 7;19(3):e0298601. doi: 10.1371/journal.pone.0298601 (PMC10919601; doi:10.1371/journal.pone.0298601)
Supplement: S1 Data — (ZIP) [file pone.0298601.s001.zip › Data/地级市Z_F工作报告.zip!/┴╔─■/│»╤⌠╩╨/└┤╫╘╣1⁄2╓┌║┼í╛┬φ┐╦╩2╛▌═°í┐_2013.docx]

各位代表：

　　市九届政府任期已经结束。现在，我代表市政府向大会报告工作，请予审议，并请各位政协委员和其他列席人员提出意见。

2012年工作回顾

　　2012年是本届政府任期最后一年，也是极不寻常、充满挑战的一年。一年来，在省委、省政府的亲切关怀和市委的坚强领导下，在市人大及其常委会的法律监督、工作监督和市政协的民主监督下，我们带领全市各族人民，坚持科学发展观，积极应对经济持续低迷下滑的严峻形势，迎难而上，奋力拼搏，收获了来之不易的成果，谱写了经济社会发展的新篇章。

　　——经济实力跃上新台阶。预计地区生产总值达到964亿元，增长15%；公共财政预算收入完成107.1亿元，增长23.2%。经济总量即将突破1000亿元，财政收入突破100亿元，这是朝阳经济发展的一次历史性跨越！

　　——面对经济下行的巨大压力，我们通过各种稳增长措施，帮助企业渡难关，有效遏制了工业税收、用电量一度下滑的不利形势，扭转了工业经济增长乏力的被动局面，基本实现了企稳回升。

　　——历经17个月艰苦鏖战，凌钢三期技改工程全面竣工，钢产能达到600万吨，凌钢成功跻身国内大型钢铁联合企业30强。

　　——成功组织了中国朝阳设施农业发展高峰论坛和市校对接、农超对接等活动，市场、育苗中心和基层服务体系建设加快推进，设施农业实现产值150亿元，增长41.6%，在农民稳定增收上发挥了重要作用。这项工作得到回良玉副总理和国家农业部的充分肯定。

　　——通过实施节水滴灌、加强大田生产服务、开展人工增雨等措施，粮食生产实现“三连增”，产量突破30亿公斤，再创历史新高。

　　——服务业增加值增速进入全省前列，县级服务业集聚区建设在全省领先，特色文化旅游产业带被评为省级重点服务业集聚区。牛河梁遗址公园、鸟化石二期工程、清风岭旅游服务中心相继落成，热水汤温泉旅游度假区、大黑山温泉小镇开工建设，凤凰山、鸟化石公园被评为国家4A级旅游景区，文化旅游硬件条件更趋完善。

　　——紧紧抓住三大需求，共组织实施投资5000万元以上项目380个，完成固定资产投资752亿元、社会消费品零售总额300亿元、出口创汇4.2亿美元。在严峻复杂的经济形势下，投资、消费和出口对经济增长的拉动作用没有减弱，而是逐步增强。

　　——“5+1”新城建设突飞猛进，新开工项目110个、面积533万平方米。燕都新城初步形成4.5平方公里建设规模，燕都商务大厦等一批重点工程实现封顶，大凌河燕都新城段治理全面完成，中心城区已形成近万亩水面和湿地。

　　——一批全市瞩目的重大基础设施工程胜利竣工。投资7.4亿元、全长53公里的中心城区外环路全线贯通，珠江大桥、锦赤铁路新线赤峰至朝阳段通车营运，新殡仪馆建成使用。机场扩建工程全面完成，朝阳—北京、朝阳—大连—烟台每日往返航班顺利通航。气化朝阳天然气工程主管线铺设基本完成，两座220千伏智能变电站建成投产。我们还投资近5亿元，拓宽改建城市道路110万平方米，提升改造中心城区上世纪50年代铺设的地下供水管道34公里。城区环境整治“百日会战”也取得丰硕成果。

　　——建筑业产值突破300亿元，增长29.8%，完成建筑业面积1652万平方米。市县两级11次大型房交会成效显著，完成房地产开发1563万平方米，实现销售收入145亿元。城中村改造、小城镇建设分别完成506万和162万平方米，增长19.3%和66.7%。

　　——大小凌河流域综合治理取得突破性进展。投资7.3亿元建设的32个水污染治理工程基本竣工，全市已形成34万吨污水日处理能力；流域内涉河尾矿库治理、河道疏浚、湿地建设取得明显成效，污水直排、私挖滥采、破坏河道等顽症得到强力整治，市人大代表连续6年关注跟踪的凌河流域污染问题得到有效解决，水质得到明显改善，朝阳母亲河正在重现生机！

　　——绿化造林和秋冬季植树战役全面展开，完成造林136万亩、城区植大树12万株、水土保持综合治理72万亩；青山工程和草原沙化治理工程扎实推进。我市顺利通过全国绿化模范城专家评审，即将成为省内第三个获此殊荣的城市。

　　——民生工程深入实施。投资8.6亿元，全面完成了向全市人民承诺的10件实事，其中新建保障性安居工程3639套、安全饮水工程745处、农村困难家庭常年病人托管中心6所，完成老旧小区改造200万平方米，农村贫困人口年人均纯收入增加到2200元以上。另外，中心城区投资4.8亿元的9所中小学建设工程全部启动，投资5.5亿元的5所医院改造工程基本竣工，小学大班额和群众就医条件得到明显改善。城镇居民人均可支配收入达到17440元，增长16%；农民人均纯收入达到9200元，增长22%，增速继续保持全省第一。

　　——面对“达维”台风引发的洪峰和近三十年最大暴雪等自然灾害袭击，我们科学研判，果断决策，夺取了防汛抢险和抗雪救灾的重大胜利，未出现人员伤亡，灾害损失也降到了最低。

　　——干事、干练、干净政府建设取得初步成效。市政府班子累计下基层1600余次，解决实际问题4300多件，组织各项工作拉练、督查150多次；通过加强政府投资项目审计监察，一批违纪违规案件得到有效查处，共节省财政资金1.6亿元，政府工作呈现出落实上见责任、推进上比效率、作风上重廉洁的新气象。

　　这里我要特别报告一下县域经济和城区经济发展变化情况。北票、建平地区生产总值突破200亿元，凌源接近200亿元，朝阳县和喀左接近150亿元，双塔、龙城两区合计达到260亿元。建平、北票公共财政预算收入超过20亿元，凌源、朝阳县分别达到13亿元和12亿元，喀左接近8亿元，双塔、龙城两区合计接近15亿元。其他各项工作也形成了多点开花的局面：

　　北票粉末冶金产业集群建设得到省政府通报表彰，产值突破200亿元，除尘装备产业集群加速发展。台吉新城建设扎实推进，完成房屋征收4000多户，金河大桥、银河大道、金河公铁立交桥等项目建成使用，新老城区实现对接贯通。全年引进内资106亿元、外资3391万美元。富贵鸟兴旺矿业、禾丰牧业熟食制品等项目竣工投产。北票金丝王大枣获国家地理标志保护产品称号。

　　凌源市荣获“中国百合第一县”称号，并被确定为国家级现代畜牧业标准化养牛示范基地和全省一县一业示范县。以九州矿业等为代表的新型建材产业集群建设取得明显进展。滨河新城新开工面积116万平方米。新引进内资103亿元、外资3395万美元，永利广场城市综合体签约落地。城市热源整合项目的实施，有效解决了困扰凌源多年的城市冬季供暖难题。

　　朝阳县有色金属园区晋升为省级经济开发区，产值突破200亿元。企业并购和股权融资工作取得新进展。全年引进内资105亿元、外资3122万美元，佰宏矿业、河南新大新材料等项目启动建设，温氏集团生猪饲养基地项目主体竣工。新县城建设强力推进，新开工面积100万平方米，行政中心、实验中学等项目实现封顶，大凌河县城段治理加快推进。郭家村城改和小城镇建设取得新突破。

　　建平红山新城建设速度居全市前列，新开工面积106万平方米，商务中心和两所中学等项目主体基本竣工。金属新材料产业集群快速起步发展，新引进内资106亿元、外资3294万美元，圣隆伟业、荣田矿业等项目竣工投产，丽佳有机杂粮、农产品物流交易市场等项目进展迅速。城区应急供水工程全面竣工，彻底结束了县城过去分时段供水的历史。

　　喀左“五城联创”已夺得国家级文明城、卫生城、园林城和省级生态县四块牌匾。河南新城已形成三纵五横道路骨架，基础设施配套和教育、卫生、体育等功能配套不断完善，通过凌河水系治理，10公里路坝结合景观带初步建成，形成水面近万亩。冶金铸锻产业链已经形成，园区被列入省级经济开发区，实现产值185亿元。新引进内资80亿元、外资2403万美元。

　　双塔中央商贸商务区被评为省政府现代服务业示范集聚区。金属新材料深加工产业集群起步发展，全年引进内资55亿元、外资1338万美元，力宝重工超纯合金、金麟超纯铁精粉等项目启动建设。都市农业快速发展，集孙家湾万亩枣园、骆驼山梨业、桃花吐设施农业示范区为一体的生态农业景观长廊初步形成。中山、孟克城中村改造完成137.3万平方米，实现了双倍增。

　　龙城现代农业园区晋升为省级经济开发区，新能源电器、矿山机械产业集群加快发展。新引进内资67亿元、外资1923万美元。万商国际城市广场、黑猫伍兴岐炭黑新生产线等项目相继开工建设，立塬电容型锂离子电池新闻发布会在北京人民大会堂成功举办。八里堡、西大营子等城中村改造完成180万平方米。紫金苑社区被授予“全国民族团结进步创建活动示范社区”称号。

　　这些新成果、新突破，让我们看到了七个县(市)区竞相发展的态势，看到了全市城乡正大踏步走进城镇化的新时代，看到了朝阳实现全面崛起的希望！

本届政府四年奋进历程

　　各位代表，过去四年，是我市在全面建设小康社会和突破辽西北进程中阔步前进的四年，是凝聚人心、真抓实干、加快发展、硕果累累的四年。四年时光虽然短暂，但我们将永远铭记这一特殊的历史时段。

　　这是突破辽西北取得重大进展、综合经济实力大幅跃升的四年。我们坚持把突破辽西北战略引入经济社会发展各领域，胜利完成了“十一五”规划，启动实施了“十二五”规划，圆满实现了“三年见成效、五年大变样”突破目标，经济持续增长，基础日益雄厚。地区生产总值、公共财政预算收入、固定资产投资分别是2008年的2.2倍、2.7倍和2.6倍，年均增长15.7%、27.8%和26.8%。县域和城区经济两大板块先后完成首轮三年倍增计划。

　　这是三次产业提速发展、结构质量不断优化的四年。凌钢三期技改、鞍凌精品钢、燕山湖电厂等一批重大项目竣工投产，工业五项工程全面实施，规模以上工业企业数量大幅增加。百万亩设施农业全面建成，质量、效益和影响力不断提升；一批10亿元以上农产品加工重大项目启动实施；累计建成畜禽标准化养殖小区2250个，畜牧业产值年均增长16%。服务业提速发展，集聚区建设扎实推进。三次产业比调整到21∶50∶29。

　　这是招商引资和项目建设深度推进、经济发展后劲有效积蓄的四年。通过开展“招商引资高峰年”、“项目年”活动，福建富贵鸟、浙江海亮、红星美凯龙等一批项目落户朝阳。四年来，共引进域外资金近2000亿元，实际利用外资5.1亿美元，出口创汇14.9亿美元，实施千万元以上重点项目2540个，分别是上个五年的3.4倍、2.8倍、1.3倍和1.7倍；销售收入超亿元民营企业由102户增加到300户，民营经济占比提高到66%。

　　这是城乡基础设施建设明显加快、生态环境全面改善的四年。我们通过争取上级扶持、土地出让收益、发行城投债券等，共完成基础设施建设和环境改造投资525亿元，是上个五年的2.1倍。“5+1”新城拔地而起，城镇化率升至43.6%。城乡道路交通网络不断完善，电网规模实现倍增。生态治理取得前所未有成效，528万亩荒山绿化全面完成，青山工程、碧水工程、蓝天工程深入实施，森林覆盖率和林木绿化率分别达到42.4%和47.8%，首次超过全省平均水平，化学需氧量和二氧化硫排放量连续实现双下降。

　　这是以民生为重点的社会建设全面展开、城乡居民得到更多实惠的四年。我们多元化筹资近300亿元，实施了一大批惠及城乡群众的民生工程，开发式扶贫、实名制就业等工作取得显著成效，城乡养老保险和基本医疗保险制度实现全覆盖，城乡居民收入大幅提高。四年来，我们连续4次提高企业退休人员养老金和城乡低保标准，累计建设保障性安居工程3.9万套，在全省率先实施了老旧小区环境改造，帮助5.6万户居民彻底告别了过去污水横流、环境脏乱差的居所。

　　各项社会事业实现长足发展，社会管理不断加强。新建和改扩建标准化幼儿园45所、中小学校舍63万平方米，七个县(市)区顺利通过省“双高普九”验收，新增省级示范高中3所，市工程技术学校、建平职教中心被确定为国家首批中等职业教育改革发展示范校立项建设学校，朝阳师专完成异地搬迁，合作办学和专升本工作扎实推进。公共卫生服务体系不断完善，医疗服务能力和水平显著提高，三所县级医院新晋升为三级医院。市县两级文化馆、图书馆、体育馆等设施日臻完善，文化体育惠民活动广泛开展，竞技体育取得新成绩，我市健儿在伦敦残奥会上夺得2金2银2铜共6枚奖牌。科技创新和人才引进步伐不断加快，科技进步贡献率达到44%。人口和计划生育各项控制指标全部完成。广播电视事业快速发展。国土资源管理水平和保障能力显著提高。平安朝阳创建得到加强，严打整治行动、安全保卫、道路交通治理取得明显成效，社会管理防控、公共安全管理、突发事件应急处理、食品药品安全体系不断完善并发挥作用，信访工作基本稳定，安全生产形势总体平稳。军民融合实现创新发展，全军首创的军民协作区建设打开双拥共建新局面，驻朝部队在饮水解困、抢险救灾中做出了突出贡献。

　　这是政府自身建设不断加强、政府行政效能显著提高的四年。我们坚持依法行政和管理创新，大力推进干事、干练、干净政府建设，持续开展下基层、抓落实、解难题活动，政府机关廉政建设和“五大系统”建设扎实推进，行政审批、财税管理、审计监督、政府采购等改革不断深化，政府工作作风、行政效率明显改善和提高。四年来市政府共办理人大代表议案、建议和政协提案878件，办复率和办结率均达100%，满意率达99.3%。

　　此外，质量监督、工商、物价、档案、住房公积金管理，民族工作和宗教事务管理，气象、水文、防震减灾、人防、援疆、外事侨务和涉台工作，以及老龄、妇女儿童、残疾人权益保障等方面也取得了新成绩。我市还先后荣获省级文明城和全国双拥模范城、全国无偿献血先进市、全国民族团结进步模范集体等称号。

　　各位代表，过去四年，我们取得了令人瞩目的成就。这一切得益于省委、省政府的亲切关怀，得益于市委的坚强领导，得益于历届政府打下的坚实基础，得益于市人大、市政协认真负责的监督和不遗余力的支持，也凝聚着全市340万人民的智慧和汗水。还应指出的是，本次大会后，市政府一些同志将离开奋战多年、感情至深的工作岗位，他们在届内尽职尽责、敢于担当、勤奋努力、真抓实干，为朝阳发展做了大量卓有成效的工作。正是有了这些，朝阳才形成了团结奋斗、不断前进的大好局面，才有了辉煌美好的今天。在此，我代表市政府，向在各个领域和岗位上辛勤耕耘的全市各族人民，向关心支持朝阳发展的老领导、老同志，向给予我们监督与支持的人大代表、政协委员、各民主党派、工商联、无党派及社会各界人士，向关心支持朝阳建设的驻朝中省直单位、解放军指战员和武警官兵，表示崇高的敬意和衷心的感谢！

　　在看到成绩的同时，我们也清醒认识到，目前朝阳仍属于欠发达地区，经济总量小，民生水平低，贫困人口多，城镇化率低，而且经济结构不合理、企业发展科技支撑不强、各类人才相对匮乏。解决这些问题将是一个长期的过程，朝阳人民艰苦创业和辛勤付出也将是一个长期的过程。除了这些制约经济社会发展的问题外，政府软环境建设、干部作风建设和廉政建设还需进一步加强，等等。对此，我们必须下定决心，在今后切实加以解决。

新一届政府奋斗目标和展望

　　各位代表，未来五年是贯彻落实党的十八大精神、全面建成小康社会的关键期，是实现朝阳全面崛起的决胜期。为了跟上辽宁率先全面建成小康社会步伐，今后五年，我们将重点实施“两步走”，即到2015年，地区生产总值实现1800亿元，公共财政预算收入实现180亿元，圆满完成“十二五”规划；到2017年，地区生产总值达到2400亿元，公共财政预算收入达到260亿元，城乡居民收入比2010年翻一番半，固定资产投资年均增长25%左右。我们要在加快推进工业化、城镇化的同时，努力实现信息化与工业化深度融合、工业化与城镇化良性互动、城镇化与农业现代化同步发展，为全面建成小康社会奠定坚实基础。

　　我们将以更大力度抓好经济结构和产业层次的提升。紧紧抓住新一轮老工业基地调整改造机遇，坚持以增量调结构，以创新促进三次产业升级。加快工业产业集群和重大工业项目建设，做大做强冶金、装备制造、农产品加工、能源及新能源等主导产业，大力培育战略性新兴产业，培育建成15个年产值超百亿元的工业产业集群，其中超千亿1个、超500亿2个、超200亿4个，并稳定形成千万吨钢生产能力。大力发展现代农业，设施农业面积达到200万亩，并努力建成以朝阳为中心的中国北方最大设施农业优质农产品生产供应基地。围绕“两高一点”目标，全面实施好服务业发展提速计划。到2017年三次产业比达到12∶58∶30。

　　我们将深入实施经济体制改革，全面提高对外开放水平。破除体制机制障碍，深化行政管理体制、财税体制、金融体制、农村土地流转和社会事业等各领域改革。完善中小微企业扶持政策，民营经济占比达到70%以上。实施更加积极主动的开放战略，以江浙招商局为前哨，通过灵活的体制机制赢取竞争优势，推动招商引资实现更大突破，利用内资、外资增速分别保持在25%和20%以上。

　　我们将完善城乡发展一体化体制机制，不断加快城镇化进程。促进户籍制度改革，有序推进农业转移人口市民化，努力实现城镇基本公共服务常住人口全覆盖，城镇化率达到55%以上。到2015年，6平方公里河东凤凰组团建设全面完成。到2017年，燕都新城10平方公里起步区全面建成，中心城市加速向“双百”规模迈进；五县(市)新城完成起步区开发建设并形成规模；加快推进省级重点镇、市级样板镇和凌河沿岸新市镇建设。

　　我们将全面推进生态文明建设，打造山更绿、水更清、天更蓝的美丽朝阳。坚持不懈地抓好绿化造林，到2017年森林覆盖率、林木绿化率分别达到46%和51%。继续抓好青山工程、碧水工程、蓝天工程，加快生态环境修复，巩固提高凌河流域综合治理成果，搞好凌河沿岸生态带、旅游带和城市带建设，推进绿色发展、循环发展、低碳发展，积极争创环保模范城和生态市。

　　我们将在改善民生和创新管理中继续加强社会建设。不断完善就业和社会保障体系、医疗卫生服务体系、公共教育体系和社会救助体系，进一步深化和创新文化惠民服务体系，加强基层政权建设和社区管理，健全基层公共服务和社会管理网络，拓宽与人民群众互动的载体和平台，努力实现更高质量的就业，实现城乡居民收入增长与经济发展同步，实现人人享有基本社会保障，实现各项社会事业发展水平的更快提高，实现发展成果更多、更公平地惠及全体人民，让人民群众生活在朝阳有幸福感和安全感，在朝阳发展有成就感，走出朝阳有自豪感！

　　各位代表，新一届政府一定要通过五年的不懈奋斗，把朝阳建成一个综合实力更强大、产业特色更鲜明、人民生活更富裕、社会建设更和谐、生态文明更彰显的城市，为朝阳今后发展赢得主动，赢得优势，赢得未来！

2013年重点工作安排

　　2013年是新一届政府届首之年。起步关系胜负，开局影响全局，做好今年工作意义重大。当前国内经济下行压力并未解除，持续时间和影响深度可能将长于预期。在这种形势下，我们既要充分估计可能遇到的各种困难和风险，做好应对的长期准备，更要坚定信心，不为任何风险所惧，抓住和用好各种机遇，努力开创朝阳经济社会发展新局面！

　　新的一年里，我们要认真贯彻落实党的十八大精神，按照市委九届四次全会暨经济工作会议的要求，继续把握好稳中求进的工作总基调，以提高经济增长质量和效益为中心，加快转变经济发展方式，创新社会管理，努力实现新一届政府扎实开局。主要预期目标是：地区生产总值增长14%，公共财政预算收入增长16%，固定资产投资增长23%，居民可支配收入增长14%以上。

一、实现工业经济持续健康稳定增长

实施有针对性的稳增长措施，抓好以企业为中心的经济运行和结构调整，加大包扶力度，协调解决企业生产经营中的实际问题，确保规模以上工业增加值增长16%。

　　全面推进工业五项工程，新增销售收入超200亿元企业1户、超10亿元8户、超2亿元25户、规模以上企业100户；全面完成省下达的并购国内外企业、节能降耗和淘汰落后产能任务。实施“专精特新”工程和创新型中小企业培育工程，加大中小微企业扶持力度，民营经济增加值增长20%以上，高新技术产品增加值增长25%以上。

　　全力支持凌钢、鞍凌钢和燕山湖电厂等重点企业抓好生产经营。加快推进国电热电联产、新都黄金、朝阳重机搬迁改造等项目开工建设。继续抓好北票30万吨铸锻件和40万吨页岩油加工、凌源整车生产、朝阳县佰宏矿产品深加工、建平富贵鸟矿业、喀左金工科技钒钛加工、双塔力宝重工超纯合金、龙城太平洋电梯等一批重点工业项目，确保实现投产达产。

二、高效推进招商引资和项目建设

　　坚持市政府主导推动、县(市)区为主的招商模式，充分发挥江浙招商局作用，以更有效的激励机制搞好招商引资，让新体制、新机制充分释放活力。坚持每月组织一次大规模、富有成效的集中招商活动，全年完成域外引资760亿元，新增100亿元；实际利用外资和出口创汇均增长20%。

　　坚持实行市政府领导联系重大项目制度，加大项目调度和要素保障力度，确保一批新引资项目尽快落地、竣工和投产，确保全年实施投资5000万元以上重点项目400个，其中亿元以上160个。突出研究好资金问题，抓好银企对接、股权投资、企业上市和二期城投债券发行工作，大力发展新兴金融业态，新增贷款、直接融资分别突破110亿元和80亿元。重点解决好项目用地、环评等要素保障问题，坚持节约集约利用土地，狠抓未利用地开发和城乡建设用地增减挂钩，实现建设用地征收与转用审批1400公顷。

　　深化财税体制改革，强化财源建设和税源监控，提高财政收入质量。加强政府投资项目招投标管理，坚决预防和遏制项目招投标中违法违规行为。

　　三、大力发展县域经济和现代农业

　　支持北票、建平加速向全国百强县冲刺，其他县(市)要在全省综合排名中继续实现位次前移。

　　加快推进工业产业集群和园区基础设施建设，北票粉末冶金、朝阳县有色金属、喀左冶金铸锻产业集群产值要分别突破260亿元、230亿元和200亿元，北票除尘装备、凌源新型建材、建平金属新材料、龙城矿山机械产业集群要加快迈入百亿元行列。

　　大力发展现代农业。深入实施设施农业提质增效工程，在搞好市场、育苗中心建设的同时，充分发挥标准化示范区引导作用，积极推进设施农业公司化运作模式，加强科技培训、信息化应用和基层服务体系建设，优化棚内种植结构，加强种子、农资质量监管，提高农产品质量安全水平，培育无公害绿色品牌。积极实施畜禽标准化、规模化养殖，因地制宜发展庭院养殖，新建标准化养殖小区400个，加强动物疫病防控。以打造经济林大市为目标，推进特色林果业发展。新引进2—3个10亿元以上农产品加工重大项目，新增1—2个一县一业示范县。

　　新增节水滴灌面积40万亩。坚持开展“大禹杯”竞赛活动，新完成水土保持综合治理面积40万亩。扩大农机作业面积，不断提高农业机械化水平。启动实施农村危房改造工程，继续抓好一事一议财政奖补村路建设工程、农村电网改造升级工程和农村环境综合整治工程。积极推进突发事件预警信息发布系统建设，科学开发云水资源，提高防灾减灾能力。

　　四、加快发展城区经济和服务业

　　把双塔工业园区作为燕都新城就业的重要承载区，加快市本级企业“退二进三”步伐，尽快做大双塔园区工业和消费性服务业规模。支持龙城新区高新技术产业和新能源产业项目建设，推动重点产业集群和生产性服务业加快发展。

　　抓好双塔中央商贸商务区、龙城现代服务业区两个百亿元集聚区建设，启动建设高水平燕都新城服务业集聚区。按照“1+2”要求，继续加快各县(市)超50亿元、超10亿元服务业集聚区建设。

　　完善新农村流通服务网络，加强城区商品市场规划建设和管理，积极引进更多的服务业新型业态、品牌和城市综合体项目，力争服务业增速高于全市经济发展水平，双塔、龙城两区服务业增速高于全省平均水平。

　　保持房地产业健康发展，房地产投资和商品房销售额均增长16%以上。创新文化旅游发展模式，通过市场化运作组建市旅游总公司，理顺管理与经营关系，深度开发文化旅游资源，组织开展文化旅游年和首届文化旅游节活动，旅游业总收入增长一倍以上。积极申报国家通用航空综合改革试点，加快通用航空产业发展。

　　五、推动新城建设提速发展

　　“5+1”新城建设要实现“三个转变、五个突破”：即由基础设施建设向城市重点项目建设转变，由政府投入向市场投入转变，由单一建设向建管并重转变，努力在重大项目、招商引资、土地出让、人口集聚、资产整合上实现新突破。

　　加快推进燕都商务大厦、万豪酒店、壹品人家精品住宅小区、市一高和一中两所新城分校等项目建设，燕都新城新开工面积达到200万平方米。北票台吉新城、凌源滨河新城、朝阳县新县城、建平红山新城、喀左河南新城要进一步加快起步区内公建、房地产、基础设施配套等项目建设，朝阳县新县城完成新开工面积200万平方米，喀左河南新城完成70万平方米，其他新城分别完成110万平方米。继续加快推进河东凤凰组团建设。全市完成建筑业面积1800万平方米，房地产开发面积1400万平方米。

　　六、抓好城乡生态文明和基础设施建设

　　深入实施青山工程、碧水工程和蓝天工程，全面完成2.4万亩矿山生态治理和坡地退耕还林收尾任务，积极推进环城绿化工程建设，新植树造林100万亩，全民义务植树1000万株，草原沙化治理40万亩；继续抓好大小凌河流域综合治理，加大面源污染治理力度，提高210座涉河尾矿库治理标准，推进32个新建污水处理工程全线运行，凌河流域水质稳定在四类标准以上；抓好工业企业脱硫、脱硝减排工程，积极推进热电联产、热源整合和集中供热，加强大气污染联防联控，完成省下达的节能减排任务。

　　加快推进气化朝阳天然气工程、凌源城区供水工程建设，确保年内竣工使用。积极配合省里搞好绥凌高速公路、京沈高铁和锦承线扩能改造、干线公路提级改造、辽西北供水工程和9座变电站等项目建设。继续搞好各县城“一路一带两广场(公园)”建设，新建和改造市县两级城市道路80万平方米，城区植大树30万株，新增绿地40公顷。全面推进城区供水、供气、供热管网改造工程。深入开展以“提升文明素质、建立长效机制”为核心的市容环境集中整治行动，做好弃管小区物业管理工作，打造文明、整洁、优美、有序、安全的生活环境。

　　七、全力抓好民生工程

　　加大对重点职业培训机构投入力度，新增实名制就业4.8万人，城镇登记失业率控制在4%以内，促进大中专毕业生特别是困难家庭大学生稳定就业、扩大就业。统筹推进城乡社会保障体系建设，建立健全乡镇社保服务网络，继续扩大各类社会保险覆盖面。

　　为人民群众办好10件实事。一是继续对设施农业大棚实施政策性保险。二是继续提高企业退休人员基本养老金标准。三是提高城镇居民基本医疗保险待遇标准。四是提高城乡居民最低生活保障和重点优抚对象抚恤补助标准。五是解决10万农村人口饮水安全问题。六是实施市县两级老旧小区改造150万平方米。七是有序解决中心城区和县城小学大班额问题。八是免除农村(含县镇)和城市涉农专业中职教育学生学费。九是通过产业扶贫、项目入户、移民搬迁等措施，确保农村贫困人口年人均纯收入达到2500元以上。十是为全市所有符合条件的缺肢残疾人免费适配假肢。

　　八、协调发展各项社会事业

　　强力发展学前教育，新建和改扩建标准化幼儿园24所；均衡发展义务教育，完成全市12所中小学建设任务；优质发展高中教育，加强标准化建设，确保3所高中通过省级示范校验收；大力发展职业教育，建立纵向衔接的应用型人才培养体系；稳步发展高等教育，加快推进朝阳师专专升本工作。继续搞好教育质量提升工程，加强师资队伍建设，改善教育设施条件，强化校园环境和校车安全管理，努力办好人民满意的教育。

　　深化医药卫生体制改革，建立和完善覆盖城乡居民的公共卫生、医疗服务、医疗保障、药品供应等体系，提高全市新农合补偿标准。完成市中心医院外科综合楼、中医院综合楼建设和四医院、卫校异地搬迁工作，新增床位2000张。加强县级医院、基层卫生医疗机构和各级医疗队伍建设，改善医疗条件，提升医疗服务水平。做好人口和计划生育工作，积极发展慈善、老龄、妇女儿童、残疾人、关心下一代等事业。

　　推动文化大发展大繁荣。加快县级博物馆、图书馆、文化馆和社区书屋等公共文化服务设施建设，实施文艺精品创作、文化志愿服务和送戏下乡等文化惠民工程，搞好文化遗产保护，加速文化产业发展，增强文化整体实力和竞争力。积极构建全民健身公共服务体系，促进群众体育、竞技体育和特色体育产业协调发展，全力做好十二届全运会排球赛承办工作。

　　九、加强和创新社会管理

　　健全完善基层社会管理体制机制，加快推进社区标准化建设，逐步提高农村“两委”工作人员待遇。认真做好信访工作，完善人民调解、行政调解、司法调解联动工作体系，解决群众合理诉求。加强流动人口和特殊人群管理服务。深化平安创建活动，加强社会管理综合治理，严密防范和依法打击各类违法犯罪活动。落实企业主体责任，强化安全生产监管，坚决遏制重特大事故发生。依法加强产品质量管理，整顿规范市场经济秩序，确保食品药品安全。认真落实党的民族宗教政策，促进民族团结、宗教和睦。强化国防教育和民兵预备役、人民防空工作，积极开展双拥共建活动。加强社会诚信建设，开展诚信教育，营造政务诚信、商务诚信、社会诚信的良好氛围。做好国家安全、统计、外事侨务、史志、档案、地震等工作。

十、建设职能科学、结构优化、廉洁高效、人民满意的服务型政府

　　坚决执行中央关于改进工作作风、密切联系群众八项规定，自觉接受人大及其常委会的法律监督、工作监督和政协的民主监督，认真办理人大代表议案、建议和政协提案，深化政务公开和政府信息公开，依法规范有序开展政府工作，不断把干事、干练、干净政府建设引向深入。

　　要从人民群众最关心的利益需求着眼，扑下身子，想事干事，让政府工作更贴近群众意愿。要求真务实，真抓实干，加大工作责任落实、过程督查、行政问责和约谈力度，盯人、盯事、盯结果。要坚持依法行政，推进行政执法制度建设，用法治思维和办法深化改革、推动发展、化解矛盾、维护稳定。

　　要加强制度建设，大力精简会议文件，开短会、讲短话，推行现场办公，不断在绩效考核、干部评价上创新管理机制，努力打造一支敢于担当、勤奋务实、雷厉风行、高效干练的公务员队伍。

　　要坚持关口前移，建立健全监察、审计、财政、司法综合联动监督机制，严格落实党风廉政建设责任制，深入推进“五大系统”建设和软环境建设，规范公共资源管理，依法查处违法违纪案件，着力解决人民群众反映强烈的突出问题。

　　我们一定要通过政府自身建设的深度推进，努力向全市人民展示一个孜孜不倦、追求创新的政府，展示一个为民、务实、清廉的政府，展示一个能够担当、能够真心为人民创造更多福祉的政府。

　　各位代表！新一届政府即将肩负使命踏上新的征程。新的一年里，我们将在市委的坚强领导下，团结和依靠全市各族人民，继续保持昂扬向上、奋发有为的精神状态，一以贯之地接力拼搏，不洗征尘地接力实干，为建设我们心中的美丽朝阳，为加快实现朝阳全面崛起和全面建成小康社会目标而顽强奋斗、艰苦奋斗、不懈奋斗！
